# Single-nucleus multi-omic profiling of polyploid heart nuclei identifies fusion-derived cardiomyocytes in the human heart

**DOI:** 10.21203/rs.3.rs-4414468/v1

**Published:** 2024-05-30

**Authors:** Sangita Choudhury, Indu Sivankutty, Youngsook Jung, August Huang, Sarah Araten, Connor Kenny, Zheming An, Ryan Doan, Floris Foijer, Erica Matsu, Ila Rosen, Jack Marciano, Asish Jain, Liang Sun, Nazia Hilal, Eunjung Lee, Christopher Walsh, Ming Chen

**Affiliations:** Boston Children’s Hospital; Boston Children’s Hospital; Harvard Medical School; Boston Children’s Hospital; Boston Children’s Hospital; Boston Children’s Hospital; Boston Children’s Hospital; Boston Children’s Hospital; University Medical Center Groningen; Boston Children’s Hospital; Boston Children’s Hospital; Boston Children’s Hospital; Boston Children’s Hospital; Boston Children’s Hospital; Boston Childrens Hospital; Harvard Medical School; Boston Children’s Hospital; Boston Children’s Hospital

**Keywords:** Heart, Cardiomyocytes, Polyploidization, Polyploid cardiomyocyte transcriptome and chromatin landscapes, Cell fusion, Somatic single nucleotide variants, Copy number variation

## Abstract

Understanding the mechanisms of polyploidization in cardiomyocytes is crucial for advancing strategies to stimulate myocardial regeneration. Although endoreplication has long been considered the primary source of polyploid human cardiomyocytes, recent animal work suggests the potential for cardiomyocyte fusion. Moreover, the effects of polyploidization on the genomic-transcriptomic repertoire of human cardiomyocytes have not been studied previously. We applied single-nuclei whole genome sequencing, single nuclei RNA sequencing, and multiome ATAC + gene expression (from the same nuclei) techniques to nuclei isolated from 11 healthy hearts. Utilizing post-zygotic non-inherited somatic mutations occurring during development as “endogenous barcodes,” to reconstruct lineage relationships of polyploid cardiomyocytes. Of 482 cardiomyocytes from multiple healthy donor hearts 75.7% can be sorted into several developmental clades marked by one or more somatic single-nucleotide variants (SNVs). At least ~10% of tetraploid cardiomyocytes contain cells from distinct clades, indicating fusion of lineally distinct cells, whereas 60% of higher-ploidy cardiomyocytes contain fused cells from distinct clades. Combined snRNA-seq and snATAC-seq revealed transcriptome and chromatin landscapes of polyploid cardiomyocytes distinct from diploid cardiomyocytes, and show some higher-ploidy cardiomyocytes with transcriptional signatures suggesting fusion between cardiomyocytes and endothelial and fibroblast cells. These observations provide the first evidence for cell and nuclear fusion of human cardiomyocytes, raising the possibility that cell fusion may contribute to developing or maintaining polyploid cardiomyocytes in the human heart.

## Introduction

During human fetal life, myocardial mass increases by the proliferation of cardiomyocytes, the principal heart muscle cells^1–5^. After birth, the ability of cardiomyocytes to proliferate decreases significantly, and with increased age, most cardiomyocytes lose their capacity to undergo cell division. Surprisingly, many of the human cardiomyocytes retain their capacity to synthesize DNA, leading to increased ploidies (sets of chromosomes)^6–8^. This polyploidization is a unique feature of human heart cardiomyocytes compared to many other species and even other mammals. Our lab and others have shown that human cardiomyocytes tend to become polyploid with age^8,9^, whereas in other species, such as fish^10^ and birds^11^, most cardiomyocytes remain diploid. The ventricular cardiomyocytes of adult mice are predominantly binucleated (80%), consisting of two nuclei (2n’2, where n denotes the haploid DNA content per nucleus). A mere 8% are mononucleated with either 2n (3%) or 4n (5%)^12^. In pigs^13^, dogs, and sheep, cardiomyocytes not only become polyploid but also show multinucleation^14,15^ (increased number of nuclei). This indicates differences in nucleation and ploidy content in cardiomyocytes between mammalian species. As we delve further into the tissue and cell makeup of organs, more examples of polyploid tissues continue to emerge, such as liver, placenta, and megakaryocytes derived from the hematopoietic, mammary tissue growth during mammalian lactation^16^ or in repairing mammalian kidney and bladder tissue^17,18^.

Polyploidization, which refers to the occurrence of more than two copies of identical or comparable sets of chromosomes, is a natural and essential part of development in different organisms and tissues across many species^19–22^. Polyploidy has been shown to confer survival advantages by enhancing cellular adaptability to environmental challenges, but also presents drawbacks, such as heightened susceptibility to aneuploidy, a condition frequently linked to genetic instability, cancer, and birth abnormalities^23–27^. Polyploidization in a cell can arise through multiple mechanisms, such as through endoreplication or endocycling, a process where DNA replication (G and S) continues uninterrupted by mitoses^28^ or as a consequence of cytokineses failure, leading to the generation of binucleate cells (endomitosis) as observed in hepatocytes, trophoblasts megakaryocytes, and larval tissues of male drosophila^29–33^. Moreover, endocycling and endomitosis are not inherently exclusive processes; in fact, both mechanisms have been observed in polyploid cells in zebrafish epicardium, where they both contribute to the increase in the number of genome copies per cell^33^.

**Figure 1:**
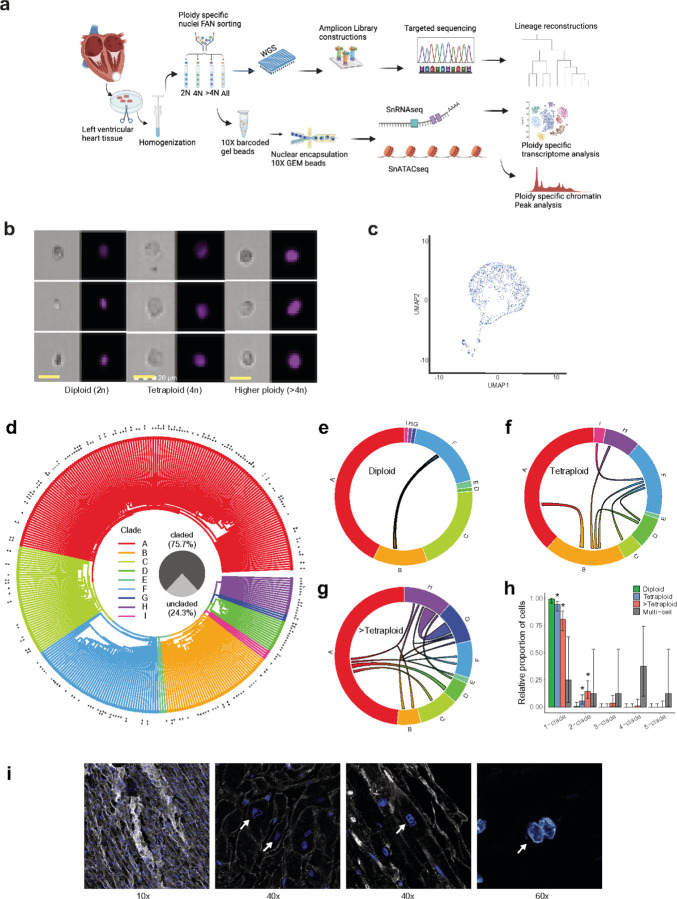
Lineage reconstruction of human cardiomyocytes via single-cell sequencing based on ploidy. (a) Schematic of approach. Nuclei are isolated from frozen postmortem heart tissues and sorted based on ploidy content by FANS. Sorted nuclei are amplified by Φ29 polymerase-mediated MDA for targeted sequencing for cell lineage analysis. (b) Representative photomicrographs from FlowSight Imaging Flow of isolated cardiomyocyte nuclei confirming DNA content of a single tetraploid and multiploid cardiomyocyte nuclei. Scale bar 20μm. (c) Purity of sorted diploid cardiomyocyte nuclei based on single nuclei RNAseq. (d) Lineage map of 340 single-clade human cardiomyocytes based on panel sequencing of 253 clonal sSNVs. Cardiomyocytes are placed into nine distinct clades defined by one or more clade-specific sSNVs. Tetraploid nuclei and >tetraploid (higher than tetraploid) nuclei are labeled by + and ++, respectively. (e-h) Cardiomyocytes generated by fusion between cells from multiple clades. The connecting arch between clades indicates nuclei containing sSNVs from more than one clade, and the thickness of arch indicates the number of nuclei. All but one diploid nuclei (e) belong to a single clade, suggesting the low double-sorting rate, whereas tetraploid (f) and >tetraploid nuclei (g) have much larger fractions of multi-clade cells generated by fusion. (h) Proportion of cardiomyocytes with different clade numbers. Tetraploid and >tetraploid cardiomyocytes show higher proportion of multi-clade cells than diploid cells (two-tailed proportion test, asterisk, p < 0.05), suggesting fusion as a mechanism for polyploidization of human cardiomyocytes. Error bar: 95% confidence interval. (i) Representative confocal images (10X, 40X, 60X) of polyploid and higher ploidy cardiomyocyte nuclei.

Aside from cell cycle alterations, polyploidization can also occur following the fusion of two neighboring cells to produce a cell with increased ploidy. Familiar examples of cell fusion include the normal development of vertebrate and Drosophila myoblasts, where immature cells formed from peripheral satellite cells fuse into myotubes through life, and mammalian osteoclasts^34–36^. Although polyploidy can arise from a variety of processes, the exact mechanism and the genomic and transcriptomic profiles of polyploid cardiomyocytes in the human heart have not been studied previously. To decipher how human polyploid cardiomyocytes are generated and how polyploid cardiomyocytes’ genome and transcriptome differ from diploid cardiomyocytes, we looked at the lineage, transcriptome, and chromatin state of polyploid cardiomyocytes in human postmortem heart tissue. We applied single-nucleus whole genome sequencing,10x Genomics single nucleus RNA-seq (snRNA-seq), and multiome ATAC + Gene expression techniques to nuclei isolated from 11 healthy hearts to comprehensively understand the origin of the human heart polyploid cardiomyocytes as well as the genomic transcriptomic repertoire. Detailed information on the sample collection is presented in Supplementary Table 1. A graphical overview of the methodology is presented in Fig. 1a.

## Results

### Cellular fusion is a significant driver of cardiomyocyte polyploidization.

To understand the contribution of cell fusion to cardiomyocyte polyploidization, we first identified developmentally acquired and clonally inherited somatic mutations that record the unique developmental lineage of somatic cells^37,38^. Using fluorescence-activated nuclei sorting (FANS), we isolated 482 single cardiomyocyte nuclei (182 diploid, 173 tetraploid, and 127 >tetraploid) from the postmortem left ventricle heart tissue of a 17-year-old individual for whom extensive insight into somatic mutations was available^38,39^. Cardiomyocyte nuclei were isolated based on cardiac troponin T, as well as by DNA content, after careful doublet exclusion^40^. These nuclei are bare nuclei without significant cytoplasm. FlowSight imaging flow analysis confirmed the ploidy and bare nuclei status (Fig. 1b), and snRNAseq on the FANS-sorted nuclei further confirmed cardiomyocyte purity (Fig. 1c). We amplified the DNA from each nucleus by multiple displacement amplification (MDA)^41^. Ultra-deep targeted sequencing for 482 MDA-amplified nuclei libraries was performed at an average depth of >7000X (Extended Data Fig. 1a), using a panel of 253 validated clonal sSNVs from the joint analysis of single-cell and bulk whole-genome sequencing (WGS) data from the same donor^38,39^. The presence or absence of clonal somatic single nucleotide variants (sSNVs) in each single-nucleus was genotyped by a Bayesian probabilistic model^42,43^ considering read counts and base quality (Extended Data Fig. 1. b, c).

Clonal sSNVs assigned 75.7% (365/482) of the sequenced cardiomyocytes into one of 9 distinct nested clades defined by the presence and absence of one or more independent mutations (Fig. 1d and Supplementary Tables 2–3), with uncladed cells likely reflecting incomplete genome amplification. Only 1/148 cardiomyocytes marked as diploid carried sSNVs from multiple clades (Fig. 1e), presumably reflecting sorting of two nuclei into the same well (doublets) and confirming that such double sorts only appear extremely rarely in our data. In contrast, 5.8% (8/138) of claded tetraploid cardiomyocytes (Fig. 1f) and 19.3% (16/83) of claded cardiomyocytes with ploidy >tetraploid (Fig. 1g) contained sSNVs from multiple clades with comparable sequencing depth and genotyping quality (Extended Data Fig. 1). We observed three- or four-claded nuclei, but only in the >tetraploid cardiomyocyte population (Fig. 1g, h). Since our study only includes bare nuclei, multi-clade nuclei can only be explained by nuclei fusion and not by endoreplication; our results suggest that at least ~5.8% of tetraploid cardiomyocytes are generated from the fusion mechanism. Further microscopic examination of >tetraploidy cardiomyocyte nuclei indicates large irregular shape nucleus in human hearts (Fig. 1i). Since our limited number of sSNV cannot detect fusion between two cells within the same clade, we estimated our sensitivity of detecting sSNVs from different clades in diploid, tetraploid, and >tetraploid cardiomyocytes separately (see Methods) and found that the proportion of cells that had undergone fusion could be up to ~13% in tetraploid cells and ~63% in cardiomyocytes with >tetraploid (Supplementary Table 3). We note that even this estimate likely understates the frequency of cell fusion due to the inherent limitation in detecting fusing cells that originate from relatively closely related origins. Thus, our data suggest that cardiomyocyte fusion is a significant contributor to the progressive polyploidization of cardiomyocytes with age.

### Single-cell multi-omics reveal gene expression and chromatin accessibility landscapes of polyploid cardiomyocytes.

**Figure 2:**
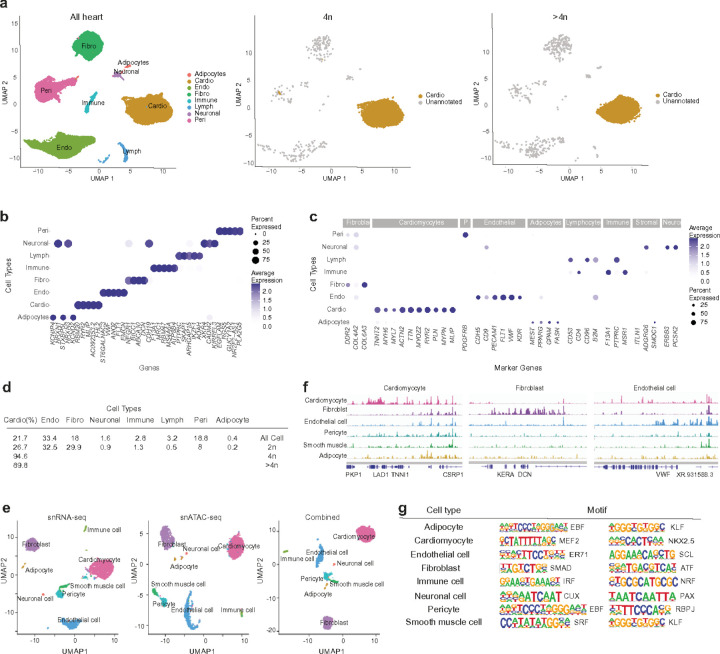
Heart cell type characterization through snRNA-seq and snATAC-seq. (a) Uniform Manifold Approximation and Projection (UMAP) clustering of 59,000 nuclei from all heart nucleic type nuclei, after QC, and Harmony integration (left panel), Tetraploid nucleus cluster middle panel, and higher-ploidy nuclei cluster (right panel). (b) Dot plots generated from the integrated dataset display five highly expressed genes in each identified population based on fold-change. (c) Dot plots showing the average expression of characteristic marker genes of each identified cell types. (d) Percent proportion of nuclei integrated into the dataset. (e) UMAP clustering of a representative Multiome dataset. Left, snRNA, Middle, snATAC, Right, combined (f) Example of cell-type-specific ATAC peaks, (g) Enriched motifs in cell-type-specific peaks.

To decipher the transcriptome and chromatin landscape of polyploid human cardiomyocytes, we performed joint snRNA- and snATAC-seq profiling on 4 postmortem left ventricular myocardium and snRNA profiling on 12 postmortem left ventricular myocardium, from 11 individuals without known heart disease (Supplementary Table 1). After quality control filtering and removal of putative doublets (Extended Data Fig. 2 and Supplementary Table 4), we obtained a total of ~59,000 human heart nuclei including ~6,000 tetraploids (4n) and ~5,200 higher-ploidy nuclei (>4n) in 19 clusters that we assigned to 8 different cell types (Extended Data Fig. 3). These included cell types like cardiomyocytes, endothelial cells, fibroblasts, pericytes, as well as rarer cell types, such as adipocytes and neuronal profiled cells (.2a-d). Consistent with published snRNA-seq studies of the human heart^44,45^ we observed cell-type-specific expression of many genes, including cardiomyocytes with high expression of *TTN, TNNT2, MYOZ2*, genes that encode force-generating sarcomere proteins and calcium-mediated processes *(RYR2, PLN*, and *MYPN)*, endothelial cells expressing *CHD5, FLT1, VWR*, fibroblast cells expressing *COL4* and *COL6* genes, lymphocytes expressing *CD96, CD53 and CD4*, immune cells expressing *F13A1, PTPRC, MSR1*, pericytes expressing *RGS5, PDGFRB*, adipocytes expressing *MEST, PPARG, GPAM* and neuronal cell types expressing *KCNIP4, NRXN1* and *PCSK2*. We also identified a list of candidate marker genes based on cell-specific expression for each of the eight cell types (Fig. 2c).

Using shared barcodes from joint multiomics profiling, we next assigned snATAC-seq profiles to the eight cell types characterized by snRNA-seq. The resulting chromatin accessibility profiles clustered by cell type (Fig. 2e) and were highly similar to those from a public human heart scATAC-seq dataset^46^, supporting the validity of our single nuclei multiome data. Peak calling performed on snATAC-seq profiles from each cell type combined into pseudo-bulk ATAC replicates uncovered a total of 686,395 chromatin accessibility peaks (Fig. 2f). These snATAC peaks were concordant among donors (Extended Data Fig. 4) and included more than 90% of peaks from a published bulk ATAC-seq study of the human heart, indicating that single-cell multiomics can recapitulate bulk ATAC-seq data. Conversely, more than half of the snATAC peaks were unique to the single-cell dataset and accessible in only one cell type. In line with this, we found snATAC peaks enriched in a cell-type-specific manner (Fig. 2f), including many located near cell-type-specific genes. With these snATAC peaks, we conducted a motif enrichment analysis to predict which transcription factors (TFs) might be active in each cell type (Fig. 2g). In accordance with published literature^47^, we observed the enrichment of binding motifs for TFs with known cell-type-specific functions, such as *Nkx2.5in* cardiomyocytes, *ER71* in endothelial cells, IRFin immune cells and *SMAD* family members in fibroblast cells. These data offer a cell-type-specific catalog of candidate TFs in the adult human heart.

### Comparing diploid and polyploid cardiomyocyte nuclei:

**Figure 3:**
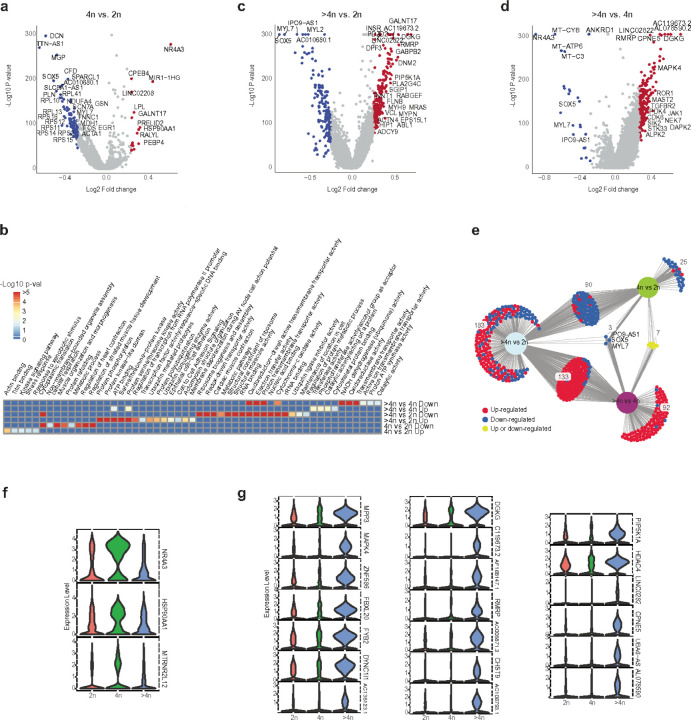
Transcriptional landscape of polyploid human heart nuclei. (a) Differential gene expression analysis between tetraploid (4n) vs. diploid (2n) cardiomyocyte nuclei. (b) upregulated and downregulated pathways in 2n, 4n and higher-ploidy (>4n) cardiomyocytes. (c) Differential gene expression analysis between >4n vs 2n cardiomyocyte nuclei. (d) Differential gene expression analysis between >4n vs. 4n cardiomyocyte nuclei. (e) DiVenn diagram indicating shared up and downregulated genes between 2n, 4n, and >4n cardiomyocytes. (f) Highly expressed genes in tetraploid and (j) higher-ploidy cardiomyocytes.

To evaluate the similarities and differences between diploid and polyploid cardiomyocyte nuclei, about 13,000 diploid, 6,000 tetraploid, and 5,200 higher-ploidy nuclei were analyzed. Differential gene expression analysis between 4n and 2n showed 125 genes that were differentially expressed (Fig. 3a): 17 were upregulated, and 108 were downregulated in 4n nuclei. Among the 17 upregulated genes (Fig. 3b), pathway analysis indicates upregulation in actin and titin binding genes, and stress response genes (Fig. 3b and Supplementary Table 5a). In contrast, many ribosomal large and small subunit genes, which play essential roles in protein translation, were downregulated. We also observed decreased expression of genes involved in mitochondrial aerobic respiration, such as *NDUFA4* and *MDH1*. Surprisingly, many of the genes regulated by *ACTC1* were downregulated along with other genes regulating muscle tissue development, such as *FOS, EGR1, SCN7A*, and *GSN*, (Fig. 3a). Specifically, *NR4A3* and *HSP90AA1* upregulation were noted only in tetraploid nuclei (Fig. 3a, f). *NR4A3* is a nuclear receptor of the subfamily 4 group A that has recently emerged as a key master gene coordinating the regulation of multiple cellular processes, including proliferation, differentiation, apoptosis, survival, inflammation, development, and lipid-carbohydrate metabolism^48^. *HSP90AA1* encodes an inducible molecular chaperone and is thought to be involved in cellular processes that are pertinent to stress adaptation^49^.

Differential expression analysis between >4n and 2n (Fig. 3c) identified 409 genes that were differentially expressed (238 genes upregulated in >4n and 171 downregulated). Among the upregulated genes (Supplementary Table 5b), pathway analysis indicates (Fig. 3b) enrichment in anatomical structural morphogenesis, which includes vinculin (VCL), an actin filament (F-actin) binding protein involved in cell-matrix adhesion and cell-cell adhesion along with *MYH9, MYPN*, and *ACTN4*. We also observed an increase in *HIP1, SGIP1, ABL1, and RABGEF1* expression. All of these genes are not only involved in the reorganization of cellular membranes but are also involved in the transport of components from a lipid-bilayer-bounded compartment to another cell and to recruit proteins essential to forming functional clathrin-coated pits^50^. Clathrin, a principal molecular scaffold, forms a lattice-like coat on and around membranes, has a particular preference for membranes enriched in phosphatidylserine and phosphoinositides and involved in the endocytosis of the transferrin receptor^51,52,53^. This complex determines much of the internal structure of a cell. In line with the findings, interestingly, we also observed the enrichment of a single KEGG pathway, the Phospholipase D signaling pathway, in high ploidy nuclei. *DGKG, MRAS, ADCY9*, and *PLA2G4C* expression were higher in higher-ploidy nuclei (Fig. 3c). Besides the cytoskeletal organization and clathrin genes, we also observe an increased expression of chromatin regulators, such as *KDM4B, HDAC4, KDM5D, and KDM2A*, in the higher-ploidy nuclei (Fig.3c, g). When we looked at the downregulated genes in higher-ploidy cardiomyocytes, we observed genes involved in cytoplasmic translation, ribosomal biogenesis, aerobic respiration, and metabolic processes (Fig. 3b, c).

Differential gene expression between higher-ploidy (>4n) nuclei and 4n identified 235 DEGs, of which 211 genes are upregulated, and 24 genes are downregulated (Fig. 3d, Supplementary Table 5c). Similar to tetraploid nuclei, higher-ploidy nuclei also indicate upregulated actin and cytoskeletal protein reorganization, increased kinase activity (Fig. 3b,d) and a down-regulation in a pathway involving oxidoreductase, cytochrome C, and transmembrane transporter activity (Fig. 3b). DiVenn analysis (Fig. 3e, Method) between diploid vs. higher-ploidy and tetraploid vs. higher-ploidy indicates 120 common upregulated genes (Supplementary Table 5d). Among the upregulated genes, we see enrichment in serine/ threonine kinases. Higher-ploidy nuclei (>4n) and tetraploid nuclei shared 80 common downregulated genes (Supplementary Table 5e) compared to 2n nuclei, suggesting that higher-ploidy cardiomyocytes downregulate many processes involving energy production. Downregulated gene-involved processes included metabolic and cellular respiration. Three genes, *MYL7, SOX5*, and *IPO9-AS1*, were downregulated in tetraploid as well as in higher-ploidy cardiomyocyte nuclei, indicating their significance in the diploid to polypoid transition of the cardiomyocyte. Downregulation of *MYL7* is expected in mature ventricular cardiomyocytes and is consistent with previous findings^54,55^. *SOX5*, a member of the *SOX* family of TFs, has a crucial function in the regulation of embryonic development, cell fate determination^56^, and cell proliferation^57^. In the heart, *SOX5* maintains heart contractility, thus downregulation of *SOX5* might reduce cardiomyocyte contractility. Importin 9 *(IPO-9)* regulates the level of actin in the nucleus^58^, downregulation of antisense Importin 9 *(IPO9-AS1)* might impact nuclear functions as actin acts as a master organizer within the nucleus^59^.

### Chromatin landscape of polyploid cardiac nuclei:

**Figure 4:**
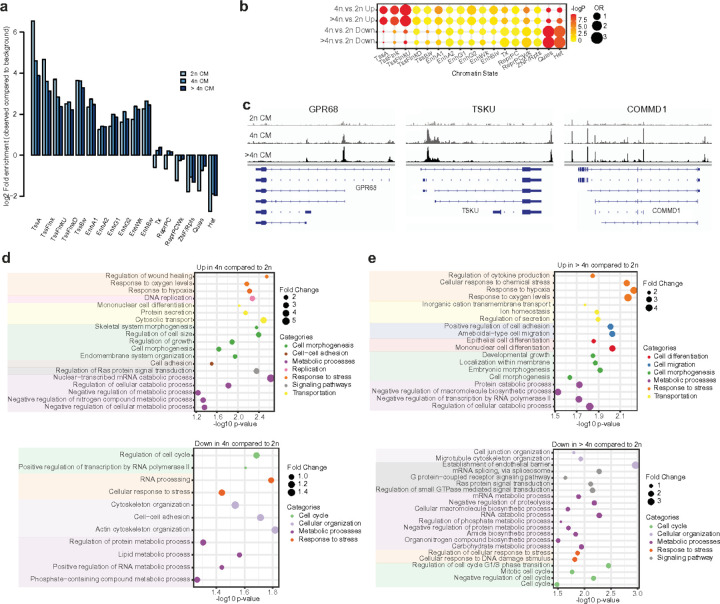
Chromatin landscape of polyploid cardiomyocytes in the human heart. (a) Chromatin state annotated based on histone modification profiles using ChromHMM Chromatin states for ATAC peaks with ploidy states. (b) Enrichment of differential chromatin accessibility with ploidy state changes in chromatin states. Enrichment for increased or decreased ATAC-seq peaks in 4n or >4n compared to 2n was calculated for each chromatin state. Odd ratios: differential peak enrichment compared to the background, Color: p-value by Fisher’s exact test. (c) Example of an increased chromatin peak for GPR68, TSKU, and COMMD1 in higher-ploidy cardiomyocytes (4n and >4n) compared to diploid cardiomyocytes. (d, e) Gene Ontology (GO) terms for genes with changes in chromatin accessibility in (d) tetraploid (4n) and (e) higher-ploidy (>4n) cell population in comparison to the diploid (2n) cell population.

Further, to investigate the changes in chromatin accessibility between diploid and polyploid cardiomyocyte genomes, we evaluated the ATAC-seq peaks that significantly change accessibility between diploid and polyploid cardiomyocytes. Accessible chromatin encompasses several key epigenome features, including active and poised regulatory regions. To verify that our ATAC-seq data correctly identifies regulatory regions throughout the epigenome, we used chromatin state annotations by ChromHMM^60^ a predictive model by a hidden markov model based on histone modifications that classifies regions of the genome into different chromatin states (e.g., heterochromatin). At first ATAC peak distribution analysis of cardiomyocyte indicates enriched active regulatory regions in our data (Fig. 4a). ATAC-seq peaks from diploid, tetraploid, and higher-ploidy cardiomyocytes were significantly enriched for the promoter, promoter-proximal regions, and enhancer regions while they were depleted for silent regions such as polycomb repressive, heterochromatin and repeat enriched regions. 2n ATAC peaks are more enriched for promoters, while tetraploid, higher-ploidy ATAC peaks tend to be more enriched for enhancers (Fig. 4a). A large number of differentially accessible peaks (both decreased and increased) was observed during the transition from diploid to higher-ploidy status. Increased ATAC peaks in 4n or higher-ploidy cardiomyocytes were significantly enriched for promoters, active enhancers, or bivalent enhancers (*p*-value < 0.001, Fisher’s exact test, Fig. 4b). Conversely, they are significantly depleted for silent regions such as weak polycomb repressive regions, quiescent, and heterochromatin regions (*p*-value < 0.001, Fig. 4b). Comparative analysis of decreased ATAC peaks between diploid and tetraploid and higher-ploidy cardiomyocytes indicates decreased ATAC peaks in tetraploid and higher-ploidy cardiomyocytes are significantly enriched for closed chromatin regions such as quiescent and heterochromatin states whereas they are significantly depleted for open chromatin regions (*p*-value < 0.001, Fisher’s exact test, Fig. 4b).

Examples of several increased ATAC-peaks from diploid cardiomyocytes to higher-ploidy cardiomyocytes can be seen in the promoter region of *COMMD1, GPR68*, and *TSKU* (Fig. 4c), which are open in tetraploid and higher-ploidy cardiomyocytes but not in diploid cardiomyocytes. The most enriched gene ontology (GO) terms for genes with increased chromatin accessibility from diploid to higher-ploidy include cellular response to wound healing, response to hypoxia, regulation of cell size, cell morphogenesis, catabolic process, and negative regulation of macromolecular biosynthesis process (Figs. 4d, e, Supplementary Table 6). In line with our transcriptomic findings, we also observed decreased peaks in protein synthesis and genomic regions involved in G2M checkpoint, *MTOR, TGF beta*, and hedgehog signaling. Similarly, we observed decreased ATAC-seq peaks in polyploid cardiomyocytes, which includes GO terms such as regulation of cell cycle, cytoskeletal reorganization, metabolic process, and stress response (Figs. 4d, e, Supplementary Table 6).

Together, these results indicate that ATAC-seq in diploid, tetraploid, and higher-ploidy cardiomyocytes can identify changes in DNA accessibility that represent key biological differences between different ploidy stages, regardless of whether these changes are due to activation/repression of specific regions within a ploidy type.

### Contrasting scRNAseq and ATACseq clustering suggests fusion between heterologous cells.

**Figure 5:**
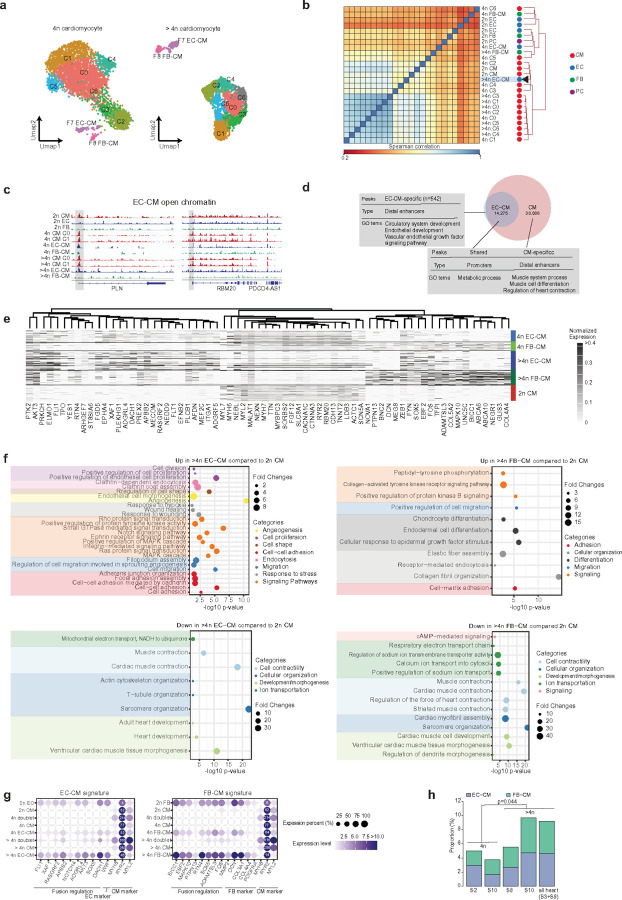
Characterizing fusion cells. (a) Unique clusters of endothelial cell (EC)-CM (purple, F7) and fibroblast (FB)-CM (pink, F8). (b) Heatmap clustering of ATAC-seq profiling of diploid and polyploid cells in the human heart. >4n EC-CM cells, pointed by an arrow, were clustered with CM-like populations. Color dots indicate cell type annotation based on expression. Red, CM-like; blue, EC-like; green, FB-like; purple, pericyte (PC)-like. (c) Examples of ATAC-seq profiles of EC-CM cells. Open chromatin of EC-CM showing promoter peaks similar to typical cardiomyocytes cells for *PLN* and *RBM20*. (d) Venn diagram showing peak comparison between EC-CM and CM (upper) and peak comparison between EC-CM and cardiomyocyte (lower) >4n cells. (e) Differentially expressed genes in EC-CM and FB-CM cells in 4n and >4n cells compared to 2n cardiomyocyte cells. Each raw represents a single cell. Expression levels are normalized to each column. (f) Pathway analysis for >4n EC-CM and FB-CM compared to 2n CM. (g) Gene expression signature for genes playing important roles in fusion, markers of endothelial, fibroblast and cardiomyocytes. Expression states for fusion cells were compared with other cell populations. (h) Quantification of EC-CM and FB-CM populations in human hearts identifying 4–10% of polyploid cardiomyocytes as either EC-CM or FB-CM.

While most high-ploidy cardiomyocytes clustered together, we observed several hundred nuclei forming unique clusters (Fig. 5a). Some of the distinct clusters--tetraploid F7 EC-CM nuclei (n = 84), and higher-ploidy F7 EC-CM nuclei (n = 124) --were identified as endothelial nuclei based on the expression signature, exhibiting high expression of known endothelial cell markers such as *VWF* and *CADH5*. Surprisingly however, ATACseq profiles of these same cells-- >4n F7 EC-CM nuclei--clustered with cardiomyocyte populations (Fig. 5b. spearman correlations between F7 EC-CM and other CM clusters are 0.71–0.87 while those between F7 EC-CM and EC are 0.25–0.44), ruling out that these cells represented technical artifacts such as double-sorting. For instance, the chromatin profile of higher-ploidy F7 EC-CM showed peaks at the promoters and enhancers of *PLN* and *RBM20* (Fig. 5c.), similar to 2n cardiomyocytes but absent in 2n endothelial cells. Thus, these F7 EC-CM populations are termed endothelial-like cardiomyocytes or EC-CM. Overall 96% of the ATAC peaks (n = 38,086) from the EC-CM population overlapped with the cardiomyocyte peaks (Fig. 5d). These nuclei also indicate ATAC peaks (n = 14,275) at cardiomyocyte-endothelial common promoters, which are involved in metabolic processes, and only 3.7% of the peaks (n = 542) were observed in distal enhancers in endothelial cell-specific distal enhancers regions, which are involved in circulatory system development and VEGF signaling pathway. Thus, the ATACseq profile of these EC-CM cells are dramatically different from typical endothelial cells, perhaps suggesting that the chromatin landscape of these cells is in the process of a cell type transition.

Differential gene expression between 2n cardiomyocyte vs. EC-CM (Fig.5e and Extended Data Fig. 5) indicated low expression of *TNNT2, ACTC1, MYH6, MYL7*, and *SCN5A* genes, and an increase in GTPase-mediated signal transduction *(PREX2, FGD5, PLEKHG1, RASGRF*, and ARHGAP) in EC-CM. We also noticed increased expression of genes associated with cell differentiation and morphogenesis, such as *AFDN, MEF2C, FLU, and ZEB1*. An increase in ephrin receptor signaling pathway genes *(EFNB2, EPHA4, and YES1)* in the EC-CM population could be linked with delayed cytokines and increased ploidy^61^. The most enriched gene ontology (GO) terms for genes with increased expression involved cell-cell adhesion, cell migration, signaling pathways (MAPK, Ras, Notch), and endocytosis (Fig. 5f left panel).

Furthermore, among non-2n cardiomyocyte populations, we also found that --tetraploid (n = 49) and higher-ploidy F8 FB-CM population nuclei (n = 99, Fig.5a) --were identified as fibroblast nuclei based on the expression signature, exhibiting high expression of collagen *(Col4A4, Col3Al)*. Differential gene expression between 2n cardiomyocytes and fibroblast-like cardiomyocytes (FB-CM) (Fig. 5e and Extended Data Fig. 5) indicated high expression of fibroblast marker genes such as *COL* and *DCN* and downregulation of cardiomyocyte genes such as *MYL2* and *RYR2*, along with upregulation of *SOX5, BICC1, EBF2* (Fig. 5e, Fig. 5f right panel). The most enriched gene ontology terms with increased expression indicate enriched genes in pathways involving cell-matrix adhesion, collagen fibril organization, receptor-mediated endocytosis, and positive regulation of cell migration (Fig. 5f right panel) such as *BICC1, EBF2, MAPK10, PTPN13* and *SOX5*. In contrast, downregulated genes are involved in sarcomere organization and cardiac muscle contraction (Fig. 5f right panel).

To evaluate whether the EC-CM and FB-CM could result from sorting of multiple nuclei from distinct cells into a single droplet, we specifically compared doublet nuclei expression profiles with the EC-CM and FB-CM populations (Fig. 5g). Doublet nuclei had very different expression profiles that did not correlate with EC-CM or FB-CM expression signatures. Potential fused EC-CM (F7) or FB-CM population (F8) indicated high expression of endothelial *(VWF)* or fibroblast *(DCN)* marker genes and downregulation of cardiomyocyte genes such as *MYL2* and *RYR2*. EC-CM cell-specific profile indicated upregulation of TF, such as *DACH1, SOX5*, and *FLI1*, and signaling pathway gene, *NOTCH4, AKT3* and *RASGRF2* (Fig. 5g), whereas the FB-CM population indicated upregulation of *BICC1, EBF2, SOX5 and* signaling pathway genes, *MAPK10, TTPN13, FOS*. Together, these results suggest a fusion-specific transcriptional landscape in higher-ploidy cardiomyocytes. Utilizing fusion-specific transcriptional markers from ploidy-specific transcriptome data, we identified 4–10% of polyploid cardiomyocytes as either EC-CM or FB-CM (Fig. 5h, Supplementary Table 7). When we transferred these fusion-specific markers to nonploidy-specific heart transcriptomic data where the nuclei were not sorted based on ploidy, we could still identify 10% of the heart cell nuclei as EC-CM/FB-CM population.

### Quantifying chromosomal copy number heterogeneity in polyploid cardiomyocyte

**Figure 6:**
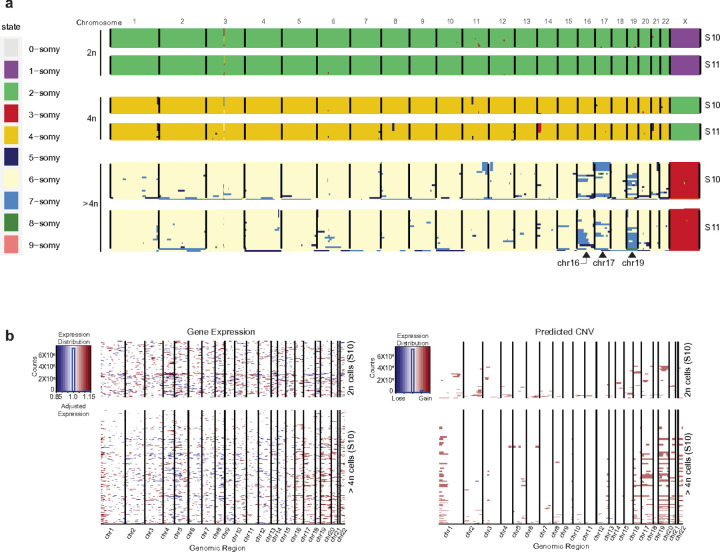
Evaluation of copy number heterogeneity for individual chromosomes in diploid (2n), tetraploid (4n), and higher-ploidy (>4n) cardiomyocyte nuclei. (a) Example of a genome-wide copy number profile of 2n, 4n, and higher-ploidy (>4n) nuclei for two individuals. Each row represents a single cell with chromosomes plotted as columns. Copy number states are depicted in different colors. Cells are clustered based on the similarity of their copy number profile. Only in higher-ploidy nuclei chromosomes 16,17,19, and 22 indicated a gain in copy number. (b) From ploidy-specific single nuclei RNAseq expression data, we evaluated changes in chromosome copy number by utilizing inferCNV. Left: The expression values for 2n are plotted in the top heatmap, and the polyploid cells are plotted in the bottom heatmap, with genes ordered from left to right across the chromosomes. The color intensities of the heatmap correspond to the residual expression values after performing a series of data transformations and effectively subtracting the 2n expression data from the higher-ploidy expression data. Right: the cells are partitioned into groups having consistent CNV patterns. CNV prediction (via HMM) is performed at the level of the subclusters and shown in the heat maps, where chromosomal region amplification shows up as red blocks and chromosomal region deletions show up as blue blocks. InferCNV analysis on the higher-ploidy cardiomyocyte also indicated a gain in copy number in chromosomes 1, 16,17,19, and 22.

Early studies on chromosomal copy number alterations related solely to disease state, but recent findings reveal that several normal human tissues, such as the human liver^62^ and fetal brain^63,64^, carry very high-levels variability of karyotypes and have been associated with enhanced resistance to stress. To evaluate whether, during polyploidization, cardiomyocytes might introduce varying degrees of gain or loss in chromosomes, we assessed the changes in copy number in cardiomyocyte nuclei. We performed single-cell whole-genome sequencing on 24 diploid, 24 tetraploid, and 24 higher-ploidy cardiomyocyte nuclei from two healthy individual donors. We detected entire chromosomes and structural aneuploidies at the single-cell level. These single-cell sequencing libraries were generated without upfront whole-genome preamplification^65,66^, enabling reliable copy number analysis based on the read density of each chromosome representing the actual copy number state. While AneuFinder^67^ (see method) did not detect chromosome copy number alterations in diploid cardiomyocyte nuclei, many of the higher-ploidy cardiomyocyte nuclei revealed copy number gains on chromosomes 16, 17, and 19 (Fig. 6a). We independently validated these findings with single-cell RNAseq data using InferCNV^68^ analysis in a larger cell number. Copy number variation analysis on higher-ploidy cardiomyocytes also indicated a gain in copy number in chromosomes 16,17 and 19 (Fig. 6b). In addition, copy number analysis of snRNAseq data indicates a gain of the short (p) arm of chr 1 and chromosome 22 in many cells. We did not observe any consistent losses in chromosomes in higher ploidy cells. On the contrary, the gain in chromosome copy number was donor-independent (Fig. 6b), indicating that copy number gain in these chromosomes could be a phenomenon of polyploidization and potentially imply a survival advantage. More in-depth study is needed to understand the role of chromosomal copy number heterogeneity in polyploid cardiomyocytes. We hypothesize that this chromosomal heterogeneity might serve as a reservoir of genetic diversity within cardiomyocytes.

## Discussion

Utilizing multiple technologies, sSNV as genetic barcode and single nuclei RNA+ATACseq of cardiomyocyte genomes from human tissue samples, this study, for the first time, showed evidence of fusion and a non-random pattern of chromosomal copy number gain in human cardiomyocytes. Our study demonstrates that polyploid cardiomyocytes can also be generated by cell and further nuclear fusion, based on clonal mutations present in individual nuclei that characterize a post-fusion cell’s genome, as we observed higher ploidy single nuclei cardiomyocyte (4n and >4n) has sSNVs that belong to more than one clade. But this study is limited in identifying fusion events only between two different clades from the endogenous sSNV lineage, and fusion of two different cell types from RNA+ATACseq. As visualization of the direct fusion events in the human heart is not a possibility, approaches utilized in this study provide us with a unique opportunity to shed light on understanding the generation of polyploid cardiomyocytes.

Previous work using the confetti model has already shown homotypic cardiomyocyte fusion in the mouse at a low level leading to multinucleated cardiomyocytes^69^, whereas, in zebrafish, cardiomyocyte fusion does not lead to increased ploidy or nucleation. Instead, it introduces cell cycle entry^70,71^. Studies have indicated that cell fusion in heart cells could have a variable outcome on the ploidy and nucleation status^72–76^. The current study is the extension of the existing phenomenon - that has previously been described in zebrafish and mice - to humans, suggesting evolutionary conservation. Identification of endothelial-like cardiomyocyte nuclei and fibroblast-like cardiomyocyte nuclei in higher-ploidy cells in the human heart suggests that polyploid cardiomyocytes can also be generated via heterotypic cell fusion and simultaneously undergo nuclear fusion. Although cell fusion events in the heart and other organs have been reported, nuclear fusion events have yet to be accepted widely. However, nuclear fusion after cellular fusion between homotypic or heterotypic cells is not a new phenomenon. Experimentally induced cells leading to nuclear fusion (for example, electrofusion and fusogenic viruses) have been shown since the 1970s^70,71^. It has also already been shown that synkaryons, cell nuclei formed by the fusion of two preexisting nuclei, can also form between cells of different types and even between cells from different species^77^, even in the absence of disease. These synkaryons can differentiate in tissue-specific ways and adopt a new fate. Others in the field also demonstrated spontaneous in vitro fusion of neonatal cardiomyocytes with endothelial cells, cardiac fibroblasts, bone marrow cells, and endothelial progenitor cells^75^. Recent evidence also indicates inflammation can act as a trigger for the fusion of myeloid-lymphoid cells with non-hematopoietic cells, including cardiomyocytes, skeletal muscle, hepatocytes, and Purkinje neurons^78,79^

This spontaneous fusion process is designed to have a protective effect and is one of the mechanisms by which damaged tissues are repaired. Certain cells may be more susceptible to fusion events due to changes in the cell membrane that are likely to happen during inflammation and the concurrent release of a range of cytokines. Since heart cells are exposed to repeated episodes of stress throughout life, fusion formation may be important in the homeostasis and maintenance of postmitotic cell types. Our previous work revealed that cardiomyocyte nuclei undergo somatic mutation accumulation faster than any other cell type in the body studied so far^9^, which might create a need for polyploidization. We hypothesize that multiple genome copies within the cardiomyocyte might provide protection against increased mutational burden and thus could have a protective effect at the cellular level^80^. Cardiomyocyte fusion during aging, disease progression, and tissue repair might be crucial to understanding the biological implications of cell fusion. Further in-depth study is required to understand the mechanism of cardiomyocyte fusion in the human heart.

## Methods

### Experimental model and subject details

Postmortem adult human hearts were procured from the NIH Neuro Bio-Bank, University of Maryland, and processed under standardized protocols under the supervision of the NIH NeuroBioBank ethics guidelines. This study was approved by the Boston Children’s Hospital Institutional Review Board (IRB, S07–02-0087). Donor information is listed in Supplementary Table1.

### Statistics and reproducibility

No statistical methods were used to predetermine sample size. The experiments were not randomized, and the investigators were not blinded to allocation during experiments and outcome assessment.

### Isolation of Cardiomyocyte Nuclei

Cardiomyocyte nuclei was isolated using previously established protocols^81,40^. Briefly, homogenization of 100mg left ventricular cardiac tissue was done in 5mL of ice-cold lysis solution containing 5 mM CaCl2, 0.32 M sucrose, 2.0 mM EDTA, 10 mM Tris-HCl (pH 8.0), 0.5 mM EGTA, 1 mM DTT (dithiothreitol) and 3 mM magnesium acetate. Using a type B pestle in a glass douncer (Sigma), the suspension was further dounced (20 strokes). The homogenized solution was centrifuged at 700g at 4 °C for 7min after filtration through 100 and 70 μm strainers (Pluriselect). The resulting crude nuclear pellets were resuspended in 2.1 M sucrose buffer containing 3 mM magnesium acetate, 2.1 M sucrose,1 mM DTT and 10 mM Tris-HCl, pH 8.0. The resuspended solution was then layered onto a cushion of 2.1 M sucrose buffer (5mL) and centrifuged for 1h at 30,000g in an ultracentrifuge at 4 °C. The supernatant was discarded and the pellet was resuspended with 1.5 ml nuclei storage buffer containing 70 mM KCl, 0.43 M sucrose, 2 mM MgCl2, 10 mM Tris-HCl (pH 7.2) and 5 mM EGTA) for further analysis.

### Flow cytometry:

For our study, accurate identification of cardiomyocyte nuclei ploidy is essential. FANS-based cardiac troponin T (Abcam, catalog no. ab ab209813; 1:250) staining and DAPI staining of the nucleus were used for isolating single cardiac nuclei on a FACSAria (100-mm nozzle, 20 psi, BD Biosciences). Cardiac nuclei were identified using a sequential gating strategy (Extended Data Fig. 6). Initially, FSC versus SSC gates were set to select the large cardiac nuclei with high SSC and FSC corresponding to more granular cells and larger cells. A combination of high FSC-H/FSC-A and SSC-H versus SSC-W plots was performed for cell doublet discrimination. Separation of the doublets from the single-cells containing 4n DNA content was done based on H versus W or A plots. Cells containing 4n DNA content will have double the A and H values, whereas W is roughly the same as cells containing 2n DNA content. The FlowSight Imaging Flow Cytometer (Luminex) has a 20x objective lens that captures the images of the nucleus and is analyzed by the IDEAS image analysis software. The software uses brightfield, DAPI to separate the cardiomyocyte nuclei. The percentage of diploid, tetraploid and higher-ploidy was determined by setting gates using the calibration with nuclei of diploid noncardiomyocytes.

### Library preparation and whole-genome amplification:

Whole genome amplification (WGA) library preparations were carried out according to the manufacturer’s instructions for the QIAseq FX single-cell DNA Kit.

### Targeted sequencing of somatic SNVs:

Amplicon primers for 253 candidate clonal sSNVs were designed for the amplicon size of 275 bp with Ion AmpliSeq Designer from Thermo Fisher Scientific. MDA-amplified cardiac nuclei were diluted 10 folds and amplicon-based enrichment was performed with pooled amplicon primers. Illumina sequencing library preparation for highly multiplexed target capture was done according to Meyer R Kircher’s protocol as described elsewhere^82^ Briefly, amplicons were generated using a high-fidelity polymerase and then were purified using a magnetic bead capture kit (Ampure; Agencourt) and quantified using a fluorometric kit (QuantIT PicoGreen; Invitrogen). The purified amplicons were then pooled in equimolar concentrations and were sequenced on Illumina HiSeq X10 with an average depth of >7000X (Extended Data Fig. 1).

### Read alignment and post-processing:

Reads generated from single-cell WGS and targeted sequencing were aligned against the GRCh37 human reference genome by BWA (ver 0.7.15)^83^ with default parameters. For single-cell WGS data, duplicate reads were masked by Mark Duplicate of Picard (ver 2.8) and then post-processed with local realignment around indels and base quality score recalibration using Genome Analysis Toolkit (GATK) (ver 3.5)^84^. For single-cell targeted sequencing data: 1) read pairs with overlapping tails were merged into single consensus reads using USEARCH (ver 11.0.667)^85^, 2) error-prone reads with gap alignment or >4 mismatches were removed, and 3) the 5bp regions at both ends of the consensus reads were masked due to its vulnerability to systematic amplification and sequencing errors.

### Somatic SNV genotyping from single-cell targeted sequencing:

The single-cell genotype likelihood was calculated for each of the 253 sSNV sites using the Bayesian model of MosaicHunter^42,43^ with three possible genotypes: homozygous for reference allele (ref-hom), heterozygous (het), or homozygous for the alternative allele (alt-hom). A minimal depth of 30X was required for confident single-cell genotyping. In each single cell, an sSNV was called if 1) the likelihood of ref-hom was <0.5; 2) ≥5 reads supported the mutant allele; and 3) the mutant allele fraction was ≥0.05. The sSNV sites that were absent in all sequenced single cells were excluded from subsequent analyses.

### Lineage reconstruction using clonal somatic SNVs:

To reconstruct the lineage tree of 482 cardiomyocytes subjected to single-cell targeted sequencing, we calculated the pairwise genetic similarity (S) as the cosine similarity of binarized sSNV profiles between two cardiomyocytes, where called sSNVs were considered as “1” and absent sSNVs were considered as “0”. Unsupervised clustering was then performed using the UPGMA (unweighted pair group method with arithmetic mean) method with the distance defined as 1-*S*. The clustered cardiomyocytes were grouped into 9 distinct nested clades (clades A-I in Fig. 1d), where each clade was defined by one or more clade-specific sSNVs (*i.e*., sSNVs present in only one clade but absent in other clades). For 93.2% (340/365) of the claded cardiomyocytes, they only harbored sSNVs from a single clade (Supplementary Tables 2, 3). In comparison, 25 cardiomyocytes (1 diploid, 8 tetraploid, and 16 hexaploid+) contained sSNVs belonging to more than one clades, suggesting their multi-clade origins that could be explained by fusion events between cells from different clades.

### Sensitivity correction for ploidy proportion:

In our sSNV-based lineage reconstruction, the detection sensitivity of cardiomyocytes derived from potential fusion events was affected by 1) whether the clade-informative sSNVs were successfully MDA-amplified and sequenced in targeted sequencing; 2) whether the origin cells were from two or more genetically distinct clades or the same clade because the latter case was not distinguishable from cardiomyocytes without fusion. To address this, we developed a mathematical model to estimate the detection sensitivity in profiled diploid, tetraploid, and hexaploid+ cardiomyocyte populations, separately. For simplicity, we only considered the fusion events involving two cells because they dominated 84% (21/25) of the detected multi-clade cells. First, the probability of capturing any clade-*i* sSNVs (*r*_*i*_) was modeled below,

$$r_{i}=\sum_{n-1}^{N}S_{in}\left(1-{\left(AD+LD\right)}^{n}\right)$$

where *N* denotes the number of sSNV in clade-*i*, and *S*_*in*_ denotes the proportion of cells in clade-*I* carrying *n* clade-informative sSNV (1 ≤ *n* ≤ *N). AD* and *LD* denote the overall allelic and locus dropout rates estimated from the genotyping profile of targeted sSNVs, respectively. *LD* was calculated as the proportion of sites with <30X depth, and *AD* was calculated as below with the assumption that reference and alternative alleles had an equal dropout chance and all alt-hom genotypes of sSNVs in single-cells resulted from allelic dropout of the reference allele,

$$AD=\frac{2P_{alt-hom}}{\left(P_{het}+2P_{alt-hom}\right)}$$

where *P*_*het*_ and *P*_*alt-hom*_ are the proportion of clade-informative sSNVs with heterozygous and alt-hom genotypes, respectively.

Next, we modeled the overall probability of detecting a fusion event by summing up all possible combinations of clades *i* and *j* after correcting for the clade-specific sensitivity due to allelic and locus dropout described previously,

$$\text{sensitivity}=\frac{\sum_{i}\sum_{j}p_{i}r_{i}p_{j}r_{j}c_{ij}}{\sum_{i}\sum_{j}p_{i}p_{j}}\;c_{ij}=\{\begin{array}{cc} 0 & i=j\\ 1 & i\neq j \end{array}$$

where *p*_i_ and *p*_*j*_ denotes the proportion of cells belonging to clade-*i* and *j*, and *O*_*j*_ is a binary value to model that a fusion event can only be detected when two origin cells are from different clades. As a result, the detection sensitivity for fusion cells was estimated as 0.21, 0.46, and 0.3 in diploid, tetraploid, and hexaploid+ cardiomyocytes, respectively (Supplementary Table 2).

### Multiomic (snRNA-seq and snATAC-seq) analysis

scRNA- and scATAC-seq libraries were generated using the 10x Genomics Single Cell Multiome ATAC + Gene Expression kit according to manufacturer’s instructions. Briefly, single cardiomyocyte DAPI-positive nuclei based on ploidy (2n, 4n, and 4n+) were sorted into sterile PCR tubes containing reagents as per the 10x user guide. GEMs were generated using the 10x Genomics Chromium Controller X and following the manufacturer’s protocol for 3’ V3.1 chemistry with NextGEM Chip G reagents (10x Genomics Inc., Pleasanton, CA, USA). The quality of the libraries generated after sc ATAC seq and sc RNAseq protocols were assessed by running them on Tapestation 4200 (Agilent Inc., Santa Clara, CA USA). The libraries were then sent to Psomagen,Inc., MD 20850 for paired-end sequencing (150 bp × 2) on a HiSeq ×10 instrument. The data from all the samples were processed systematically by doing demultiplexing on raw data, aligning to GRCh38 Ensembl (hg38) followed by quality control and filtering (Extended Data Fig.2). We used cellranger 6.1.1, and cellranger-arc 2.0.0 to pre-process snRNA-seq and Multiome data. We used an in-house scRNA-seq pipeline that was built based on the Seurat R package (R v4.2.3, Seurat v4.3.0)^86^ to carry out the downstream analysis including quality control, cell filtering, spectral clustering, cell type annotation, differential gene expression, and visualization of the scRNA-seq data. We used DoubletFinder (v2.0.3)^87^ to identify and filter out doublets from the data. We further filtered out cells exhibiting extremely low or high library sizes and number of gene features, falling outside the 95% confidence interval, as well as those displaying high mitochondrial content (above the 10%). Cells of good quality were integrated using harmony (v0.1.1)^88^ and data dimension reduction was carried out using the top 30 harmony dimensions and clustered using FindClusters function with a resolution of 0.5. After the cell clusters were determined, their top marker genes were identified with the FindAllMarkers function, and subpopulations of cells were identified using well-known markers (Extended Data Fig.3). For differential expression analysis, we used FindMarkers function from Seurat with default parameters, which is the Wilcoxon rank sum test with logfc.threshold = 0.25 and min.pct = 0.1. We used DiVenn (https://divenn.tch.harvard.edu/v2/)^89^ to compare differentially expressed genes for ploidy comparisons. It visualizes the unique and common genes between ploidy comparisons in the form of networks. The upregulated and downregulated genes are marked as red and blue, respectively. The genes that are upregulated in one comparison and downregulated in others (or vice versa) are marked as yellow. FRIP (Fraction of fragments in promoters or peaks) scores and promoter enrichment scores were used for quality control of ATAC-seq data (Extended Data Fig. 4). Cell Ranger ARC (10x genomics) and Seurat+Signac (Satija lab) were used for sn ATAC-seq data analysis.

### Quantification of the copy number variations in polvploid cardiac nuclei

We evaluated the heterogeneity in copy number for individual chromosomes in diploid (2n), tetraploid (4n) and higher-ploidy (>4n) cardiomyocyte nuclei. Single cardiomyocytes were sorted into 96 wells and 384 wells containing appropriate buffers and sent for library generation and sequencing at the European Research Institute for the Biology of Ageing (ERIBA), 9713 AV Groningen. We sequenced 24 single cell libraries from two cases for each sample (2n, 4n and >4n) and analyzed all samples with the standard version of AneuFinder^67^. Aneuploidy was measured as the divergence of a given chromosome from the diploid state utilizing AneuFinder from ploidy-specific single-sorted nuclei. Single-nuclei sequencing reads were aligned and counted in non-overlapping bins of variable size based on mappability. The hidden Markov Model with multiple hidden states was applied to the binned read counts to predict the copy number state of every single bin. At the cell population level, heterogeneity is measured as the number of cells with a distinct copy number profile within the population. In addition, we also performed copy number variations analysis using the single-cell RNAseq data on the higher-ploidy cardiomyocyte nuclei. We employed inferCNV (v1.14.2)^68^, a computational tool designed to infer copy number variations from single-cell RNA sequencing data, to investigate genomic instability in the higher-ploidy cardiomyocyte nuclei across different cell types using default parameters.

### Immunostaining

Immunostaining was performed using a standard protocol. Briefly, fresh-frozen left ventricular human heart tissue was embedded in OCT and cryosectioned (10–12μ). Sliced were stored at −80°C until further use. The sections were fixed for 30 min with ice-cold methanol, followed by antigen retrieval in citrate buffer pH 6.0 (Millipore Sigma) for 20 min and permeabilization using 1% donkey serum in PBS plus 0.5% Triton X-100. After putting the sections in blocking buffer (PBS-T containing 5% donkey serum) for 30 min at RT. The sections were rinsed again in PBS-T and then stained for WGA (Wheat Germ Agglutinin) and DAPI at 1:1000 dilutions for 30 min and imaged on a LSM 980 confocal microscope. The Super-resolution airy scan images were also imaged on the same microscope.

## Data Availability

Transcriptome and genome data will be deposited in the NCBI dbGaP with accession number phs002284.v1.p1. The data are available under controlled use conditions set by human privacy regulations. Other data are available upon request.
